# Principles and procedures for data and safety monitoring in pragmatic clinical trials

**DOI:** 10.1186/s13063-019-3869-3

**Published:** 2019-12-09

**Authors:** Gregory E. Simon, Susan M. Shortreed, Rebecca C. Rossom, Robert B. Penfold, Jo Ann M. Sperl-Hillen, Patrick O’Connor

**Affiliations:** 1 Kaiser Permanente Washington Health Research Institute, Seattle, WA USA; 2 HealthPartners Institute, Minneapolis, MN USA

## Abstract

**Background:**

All clinical trial investigators have ethical and regulatory obligations to monitor participant safety and trial integrity. Specific procedures for meeting these obligations, however, may differ substantially between pragmatic trials and traditional explanatory clinical trials.

**Methods/Results:**

Appropriate monitoring of clinical trials typically includes assessing rate of recruitment or enrollment; monitoring safe and effective delivery of study treatments; assuring that study staff act to minimize risks; monitoring quality and timeliness of study data; and considering interim analyses for early detection of benefit, harm, or futility. Each of these responsibilities applies to pragmatic clinical trials. Just as design of pragmatic trials typically involves specific and necessary departures from methods of explanatory clinical trials, appropriate monitoring of pragmatic trials typically requires specific departures from monitoring procedures used in explanatory clinical trials. We discuss how specific aspects of pragmatic trial design and operations influence selection of monitoring procedures and illustrate those choices using examples from three ongoing pragmatic trials conducted by the Mental Health Research Network.

**Conclusions:**

Pragmatic trial investigators should not routinely adopt monitoring procedures used in explanatory clinical trials. Instead, investigators should consider core principles of trial monitoring and design monitoring procedures appropriate for each pragmatic trial.

## Background

Design elements of pragmatic clinical trials intend to improve both the efficiency of generating clinical evidence and the relevance of that evidence to real-world practice. Pragmatic trials may depart from traditional or explanatory clinical trials in various respects, such as inclusion of more heterogeneous participants from typical practice settings, greater flexibility and variability in delivery of interventions, and ascertainment of outcomes from “real world” data sources [1–3]. Regardless of these design differences investigators leading both explanatory and pragmatic clinical trials have the same fundamental ethical obligations to monitor the safety of trial participants, the risks of study treatments, the integrity of trial data, and the likelihood that continuing a trial may not yield a definitive result. Design features of pragmatic trials will often influence how investigators, independent safety monitors, and independent monitoring committees assure that those ethical obligations are met.

In 1998, the National Institutes of Health (NIH) recognized and specified obligations for monitoring integrity and safety in all clinical trials funded by NIH [4]. Since then, trial investigators have been required to create specific data and safety monitoring plans. Depending on the size of the trial, the potential risks of the treatments or interventions under study, and the need for blinded analyses of interim results, responsibility for monitoring might belong to the principal investigator, an independent safety monitor, or an independent Data and Safety Monitoring Board (DSMB). Whether or not an independent DSMB is necessary, appropriate monitoring of participant safety and trial integrity is expected to include:

- Monitoring recruitment to assure enrollment of a sample sufficient to answer the study question
- Monitoring safe and effective conduct of treatments delivered to study participants
- Assuring that the study protocol and the actions of study staff minimize risks to participants
- Monitoring the timeliness and quality of study data
- If necessary, recommending early conclusion of a trial when significant benefits or risks have been demonstrated OR when the trial is unlikely to achieve sufficient precision or sample size

Pragmatic clinical trials typically differ from explanatory clinical trials in several dimensions [1, 3, 5]. Participants may be enrolled in the course of routine health care delivery. Comparisons more often involve treatments in widespread use, rather than new treatments with potential for unknown risks. Treatments are often delivered by community providers, with less investigator control over treatment quality or fidelity. Treatment exposures and outcomes may be assessed by community providers or extracted from health system records rather than directly measured by study staff. Study questions may focus on longer-term outcomes that accumulate over months rather than days or weeks.

These characteristics of pragmatic trials may influence data and safety monitoring, but investigators in pragmatic trials have the same ethical and regulatory obligations to monitor participant safety and trial integrity. Each of the five monitoring obligations listed above still applies. Different processes, however, may be necessary to meet those obligations [6]. Just as the design and conduct of pragmatic trials require specific departures from the procedures of explanatory clinical trials [1, 3, 5], data and safety monitoring for pragmatic trials may require specific departures from traditional data and safety monitoring procedures.

We describe below how data and safety monitoring procedures in pragmatic trials must often differ from those of explanatory trials. We organize that discussion around the five specific monitoring obligations described in NIH policy. For each obligation or monitoring question, we provide examples of monitoring procedures from three ongoing pragmatic trials conducted by our Mental Health Research Network [7–9]. Brief descriptions of those three example trials are shown in Table 1.

**Table 1:** Pragmatic trial examples

**Reducing Cardiovascular Risk in Adults with Serious Mental Illness (SMI Wizard)**	
• Design: Cluster-randomized pragmatic trial of decision support to reduce cardiovascular risk factors in adults with serious mental illness (SMI; psychotic disorder or bipolar disorder)	
• Setting: two integrated health systems	
• Participants: Adult outpatients aged 18 to 75 years who have SMI, have at least one cardiovascular risk factor not at goal at index visit, and make at least one follow-up visit to primary care	
• Intervention: Provider-facing and patient-facing decision support to identify, prioritize, and address specific cardiovascular risk factors	
• Comparator: Usual care (no decision support is provided, but no care is withheld or restricted)	
• Outcome: Reduction in Framingham 10-year cardiovascular event risk score	
• Safety monitoring plan: Local safety monitors reviewed any feedback on the clinical decision support provided by primary care providers during the intervention. A three-member DSMB reviewed study progress (including blinded review of interim analyses) twice per year. Safety outcomes assessed included differences between intervention and control groups in suicidal ideation, suicide attempt, psychiatric hospitalization, or emergency department visit rates.	
• Registration: ClinicalTrials.gov NCT02451670	
• Status: Enrollment, intervention, and outcome ascertainment completed (*n* = 8937); data analysis in progress	
**Safer Use of Antipsychotics in Youth (SUAY)**	
• Design: Cluster-randomized pragmatic trial of multi-component intervention to reduce unnecessary antipsychotic medication use in children and adolescents with non-psychotic disorders	
• Setting: Three integrated health systems and one pediatric health system	
•Participants: Outpatients aged 3 to 17 for whom a mental health specialty or primary care provider initiates a prescription for antipsychotic medication in the absence of a diagnosis of psychotic disorder, mania, autism spectrum disorder, or intellectual disability	
• Intervention: Intervention program including:	
○ Decision support at time of initial prescription recommending alternative treatments	
○ Pro-active expert consultation to prescribing provider	
○ Care navigation for patients and families to promote and facilitate non-pharmacologic treatments	
○ Expedited access to psychosocial treatment via videoconferencing	
• Comparator: Augmented usual care (decision support only without consultation or care navigation)	
• Outcomes: Proportion using antipsychotic medication 6 months after enrollment AND person-months of antipsychotic medication use during 6 months after enrollment	
• Safety monitoring plan: Data regarding hospital admissions, emergency department visits, and self-harm diagnoses are extracted from health system records. A National Institute of Mental Health standing DSMB reviews study progress (including blinded interim analyses regarding potential harm) three times per year	
• Registration: ClinicalTrials.gov NCT03448575	
• Status: Enrollment in progress (401 enrolled as of April, 2019)	
**Suicide Prevention Outreach Trial (SPOT)**	
• Design: Patient-randomized pragmatic trial of two population-based outreach programs to reduce suicide attempt or self-harm in adults at elevated risk	
• Setting: Four integrated health systems	
• Participants: Adult outpatients reporting frequent thoughts of death or self-harm on routinely administered depression questionnaires	
• Interventions: Two outreach interventions provided as supplements to usual care	
○ Outreach, risk assessment, and care management delivered primarily via online messaging	
○ Online training in Dialectical Behavior Therapy supported by online coaching	
•Comparator: Usual care (participants are not contacted, no care usually available is withheld or restricted)	
• Outcomes: Self-inflicted injury or poisoning over 18 months following randomization	
• Safety monitoring plan: Local safety monitors review study team responses to risk assessments indicating high risk or urgent need. National Institute of Mental Health standing DSMB reviews study progress (including blinded review of interim analyses) three times per year	
• Registration: ClinicalTrials.gov NCT02326883	
• Status: Enrollment completed (*n* = 18,887); intervention delivery and outcome ascertainment ongoing	

### Monitoring recruitment or enrollment

For either pragmatic clinical trials or more traditional explanatory trials, monitoring of recruitment or enrollment addresses the same fundamental question: Will this trial enroll a sufficient number of participants to yield a credible and reliable result? Addressing that question requires monitoring the rate of enrollment and comparison to original expectations. Monitoring may also include comparing the clinical characteristics of those enrolled to expected characteristics (especially characteristics related to primary study outcomes) and comparing actual outcomes or event rates in participants assigned to a control or usual care group to the anticipated outcomes or event rates previously used to estimate sample size. Procedures for this monitoring would usually differ little between pragmatic clinical trials and explanatory trials.

Responses to slow or inadequate enrollment, however, may differ between pragmatic trials and explanatory clinical trials. Rather than relying on volunteers or referrals, pragmatic trials may automatically enroll all eligible patients served by participating providers, clinics, or health systems. Population-based enrollment intends to increase both efficiency of trial enrollment and representativeness of trial participants. Eligible participants may be identified directly from data in electronic health records. One fortunate consequence of this population-based recruitment is that trial eligibility or enrollment is often more predictable or stable [10]. Investigators can apply trial eligibility criteria to historical records data to accurately estimate the number or rate of eligible patients in each study site [10]. One unfortunate consequence of this procedure, however, is that investigators or study staff have fewer options for increasing enrollment if actual experience falls short of prediction. When all eligible participants in a defined population are identified or enrolled automatically (e.g., they meet inclusion/exclusion criteria defined in electronic records) rather than actively recruited (e.g., through advertisements or outreach to treating clinicians), investigator or study staff effort will have no effect on enrollment rate. Increasing enrollment will instead require extending the enrollment period, relaxing eligibility criteria, and/or including additional providers, clinics, or health systems. A pragmatic trial DSMB observing an inadequate rate of enrollment may, therefore, be quicker to recommend adding study sites rather than waiting in hope that more vigorous advertising or outreach at existing study sites will yield greater returns.

Cluster-level randomization is a common feature of pragmatic trials and can create an additional layer of complexity in assessing the likelihood that recruitment will yield an adequate sample size. Statistical power or precision depends not just on the number of patient participants, but also on the number of clusters randomized, the distribution of cluster sizes, and the correlation of outcomes within those clusters [11, 12]. While some of these may be measurable in advance, cluster randomization creates the potential for imbalance in cluster size or imbalance in within-cluster correlation between randomization groups. Consequently, monitoring of enrollment in cluster-randomized trials may require more than simply plotting observed number of patients enrolled against expected.

For example:

- Given the cluster-randomized design of the Safer Use of Antipsychotic in Youth (SUAY) trial, achieving sufficient precision or statistical power depends not only on the number of patients enrolled, but also on the distribution of number of participants per provider. Consequently, the study team and sponsor monitor the number of patient participants enrolled in the intervention and usual care groups as well as the clustering of participants under providers in each group.
- Suicide Prevention Outreach Trial (SPOT) power and sample size calculations were based on an expected 3.7% rate of suicide attempts or self-harm events in outpatients reporting frequent thoughts of death or self-harm [7, 10]. Monitoring of enrollment included monitoring of event rates in the usual care control group to re-evaluate necessary sample size prior to completing enrollment.

### Monitoring safe and effective delivery of study treatments

Monitoring delivery of study treatments typically considers two questions. The first involves detection of adverse effects, especially previously unrecognized adverse effects of new treatments. The second involves fidelity or quality of treatment delivery.

Regarding detection of adverse effects, explanatory clinical trials often involve new treatments, where experience is limited, and discovery of unknown adverse effects is an important goal. That discovery could involve surveillance for any rare or unexpected events in patients receiving a new treatment. It could also involve comparing rates of more common events in patients receiving that new treatment to rates in those receiving a standard treatment or placebo. When monitoring for unexpected events, any inference regarding the relationship to study treatment often depends on the clinical judgment of study staff, investigators, or independent monitors. Comparisons of rates between randomization groups typically requires interim analyses of timely data from blinded research assessments.

In contrast, pragmatic trials often involve treatments in widespread use, where safety profiles are well established. Any apparent signal of a previously unknown adverse effect must be weighed against prior experience. If the cumulative sample size of previously completed trials far exceeds a planned new trial, then a new and unexpected “signal” of an adverse effect is more likely to be a chance finding. Consequently, attempts to identify previously unrecognized risks of study treatments are expected to have lower value in pragmatic trials involving well-studied treatments or treatment components.

The procedures employed to identify or quantify adverse effects in explanatory clinical trials may be impractical in pragmatic trials. When study outcomes or potential adverse events are ascertained from health system records, access to data is often delayed, and clinical detail may be limited. For some hospitalizations, only insurance claims data (limited to diagnosis and procedure codes) may be available to the research team. Timely review of clinical data to assess “relatedness” of adverse events to study treatments may not be possible.

For more common adverse effects, valid inference regarding relatedness requires comparison to an untreated control group. In many pragmatic trials, contact with participants is unequal across treatment arms. If ultimate trial outcomes are ascertained from health system records, study staff may have no contact with participants assigned to a usual care control group. If contact with study staff differs markedly between treatment arms, between-group comparison of adverse events reported to or discovered by study staff would certainly be biased.

Monitoring for adverse effects should also consider variable intervention fidelity or adherence and risks in specific clinical populations. Adverse effects of study treatments may be more often observed when or where those treatments are delivered most vigorously. Patients enrolled in pragmatic trials may differ from those in previous explanatory trials, so new adverse effects may still emerge.

For example:

- In the SMI Wizard trial, decision support recommendations may prompt prescribing of antihypertensive medications, lipid-lowering medications, or medications to aid in smoking cessation. While any of these medications may have adverse effects, those adverse effect profiles are well established through large clinical trials and decades of clinical experience. Consequently, the data and safety monitoring plan does not include any ascertainment of or analyses regarding adverse effects of those medications.
- In the SPOT trial, study staff have more frequent contact with participants assigned to the care management intervention, less frequent contact with participants assigned to the skills training intervention, and no contact with those assigned to the usual care control group. While study staff may learn of suicidal behavior during these contacts, any between-group comparison of rates of suicide attempts reported to study staff would be irretrievably biased. Consequently, the data and safety monitoring plan does not include any comparison of incidentally discovered suicidal behavior.

Regarding effective delivery of interventions, the relevant question for any clinical trial is whether differences in treatment exposure between groups support valid inference, especially in the event of a null result. A finding of no difference between treatment groups may be uninterpretable if the difference in actual treatment exposure is too small [13]. Explanatory clinical trials typically intend to assess or compare efficacy of treatments when optimally delivered [5, 14]. Consequently, monitoring of those trials should attend to the quality or fidelity of treatment delivery and take appropriate corrective action when quality deviates too far from the optimal. Pragmatic trials do allow, or even embrace, variable uptake and imperfect fidelity [1, 5] because this variation increases external validity study. Acceptability and ease of use are central components of effectiveness. But there are limits to pragmatism, and monitoring should consider credible boundaries for uptake or adherence. Failure to deliver intervention components with minimally adequate fidelity or adherence may undermine valid hypothesis testing. Consequently, explanatory clinical trials and pragmatic trials might use similar methods or procedures for monitoring treatment quality or fidelity but have different thresholds for intervening when fidelity or quality fall short of expected [6].

For example:

- In the SMI Wizard trial, decision support recommendations are automatically delivered to clinicians through the electronic health record. Clinicians, however, may not attend to those recommendations or share them with patients (a key component of the intervention). Consequently, the study team monitors the rate at which patient-focused recommendations are printed as an indicator of actual delivery of intervention recommendations.
- In the SPOT trial, participants are randomly assigned to the offer of outreach intervention or to usual care, with the expectation that many offered intervention services will decline or not respond to that offer. Pilot work found that approximately 40% of those offered either outreach intervention chose to fully engage. Consequently, the data and safety monitoring plan included tracking of this engagement rate at all sites to assure that actual engagement was similar to that 40% benchmark rate.
- The SUAY trial monitors the rate at which treating clinicians scan consultation recommendations from the study psychiatrist into the patient’s medical record as an indication that clinicians actually received consultants’ recommendations.

### Minimizing risks

Assuring that investigators and study staff act to minimize risk is distinct from monitoring adverse effects of study treatments. The latter question concerns inference or causation (Does the study treatment cause harm?), while the former concerns potential conflict of interest (When a risk or urgent need is discovered, do researchers place duty to participants over duty to the study protocol?). Attention to that potential conflict of interest is a fundamental responsibility of investigators and independent safety monitors in both explanatory and pragmatic trials. Lower levels of control over treatment in pragmatic trials, however, may limit investigators’ or monitors’ ability to identify or reduce risk to participants.

In explanatory trials, investigators or research clinicians are often directly involved in delivery of study treatments and monitoring of treatment effects. In pragmatic trials investigators and other study staff are often more removed from clinical care provided to study participants. Consequently, any actions to minimize risk are limited by how rapidly and accurately researchers can identify risk, what clinical actions study staff can take directly, or how well study staff can communicate risk information to treating clinicians.

For example:

- In the SPOT trial, messages to study staff may identify participants at high risk of suicidal behavior (e.g., suicidal ideation with plan and intent). In those cases, study staff attempt immediate outreach to assess risk and facilitate appropriate follow-up care, including urgent or same-day evaluation. Outreach attempts and follow-plans are documented, reviewed by a safety officer at each site for adequacy, and regularly reported to the study’s DSMB. While this outreach and facilitation of follow-up could blur intended distinctions between the two interventions, investigators and study staff are ethically obligated to respond appropriately to urgent clinical need.
- In the SMI Wizard trial, recommendations to use psychotropic medications less likely to promote weight gain could precipitate worsening of mood or psychotic symptoms. Consequently, safety monitoring includes identification of psychiatric hospitalizations and emergency department visits and assessment for appropriate management of these events.

### Data quality and timeliness

The integrity and utility of any trial result depend on the integrity and timeliness of data regarding the trial outcome(s). In explanatory trials, the research team often controls the entire chain of custody for outcome data. Outcomes are ascertained or measured by study staff, recorded in a study-specific database, and transmitted to a study data coordinating center. Monitoring the integrity and quality of study data typically involves auditing of case report forms and validation of the study database against original records.

In many pragmatic trials, outcome data are extracted from records generated by routine health care operations [1, 5, 15]. Researchers do not control every step in the chain of custody for those data. This separation of researchers from data generation and collection reduces the chance that researchers’ expectations or preferences will influence assessment of outcomes. But it allows for other sources of error. Data systems may change, or recording may be influenced by changes in business processes or EHR environment [15]. Apparent changes in study outcome rates could be artifacts of changes in data systems or indicate true changes in practice patterns. Monitoring of data quality includes both awareness of those potential disruptions and monitoring trends in outcome data streams for discontinuities [6, 16].

In explanatory clinical trials, monitoring of data integrity sometimes includes following the audit trail from an analytic database back to original clinical documents. When study data are extracted from health systems’ clinical or administrative records, obligations to protect patient privacy or health system proprietary information [17] may preclude direct access to original or primary data sources.

For example:

- The SPOT trial began prior to participating health systems’ transition from use of ICD-9 to ICD-10 diagnosis codes. Given significant changes in coding of self-harm or suicide attempt, the study team carefully examined implementation of new diagnosis codes in health system EHRs and closely monitored system-wide trends in rates of self-harm diagnoses around the transition from ICD-9 to ICD-10 [18]. That monitoring showed a reassuring stability of population-level rates for the primary study outcome (injuries or poisonings coded as self-inflicted or having undetermined intent).
- The SMI Wizard trial relies on blood pressure measures collected in routine care. Measurement procedures are less standardized than in research settings and may vary across primary care clinics. Use of digital BP measurement devices may reduce variation in quality of blood pressure measures. Consequently, monitoring of data quality and integrity included balancing methods of blood pressure measurement across study arms and monitoring clinics for systematic changes in measurement procedures or equipment.

### Early detection of benefit, harm, or futility

In explanatory trials, DSMBs review interim analyses to determine whether available data either clearly demonstrate a clinically meaningful difference (in benefit or risk) between treatments or clearly indicate that a clinically meaningful difference will not be detected within the proposed study sample size or follow-up period [19]. The ethical obligation to terminate study enrollment or delivery of study treatments sooner than planned is clearest when interim analyses indicate that continued enrollment would assign participants to a treatment already shown to have either inferior benefit or greater risk than an available alternative. That central obligation does not differ between explanatory and pragmatic trials. Plans for interim analyses in pragmatic trials must consider both practical and ethical differences from explanatory trials.

On the practical side, reliance on outcome data extracted from health system records means that data regarding benefits or harms may not be available for months after positive or negative outcomes occur. Consequently, the likelihood of detecting a clinically important difference between treatments prior to completion of enrollment may be small. Pragmatic trials may be concerned with downstream costs of alternative treatments. Premature determination of futility may preclude accurate assessment of costs or other downstream effects.

In addition, the ethical implications of terminating enrollment in a pragmatic trial may be different from those in an efficacy trial. Pragmatic trials often focus on implementation or policy questions, and a “policy-meaningful difference” is harder to define than a clinically meaningful difference [6]. Implementation decisions must consider magnitude of benefit, anticipated cost, and competing health system priorities. Consequently, early termination of a pragmatic trial as soon as the benefit of a new program or service exceeds a boundary of statistical significance does not necessarily guarantee that health systems will adopt or implement that new program. A larger sample or longer period of observation may be necessary to assess costs or broader benefits relevant to implementation decisions. In contrast, any evidence that a new program or service produces significant harm would certainly justify premature termination of trial enrollment or intervention delivery. Consequently, rules or boundaries for premature termination in pragmatic trials may often be asymmetric—more likely to stop for early indication of harm than for early indication of benefit. In cluster-randomized trials, interim analyses should appropriately account for clustering of outcomes within randomization units and attend to the possibility of bias due to imbalance between clusters.

For example:

- The SUAY trial hopes to demonstrate that a multi-component intervention will reduce unnecessary prescription of antipsychotic medication to children and adolescents with non-psychotic disorders. Interim analyses, however, focus on the possibility that reducing use of antipsychotic medication might cause harm. Those analyses will compare intervention and control groups on potential indicators of clinical decompensation: psychiatric hospitalization, emergency department psychiatric care, and self-harm or suicide attempt diagnoses.
- The SPOT trial is examining whether either of two outreach programs reduces the risk of fatal or non-fatal suicide attempt, with a sample size adequate to detect a 25% reduction in risk. Given that outcomes may occur over up to 18 months after randomization, it is not plausible that such an effect would be detected before the last participant was randomized. In addition, premature termination of enrollment or intervention would not offer any immediate benefit to those who would be assigned to usual care. But the data and safety monitoring plan includes interim analyses to detect the possibility that either intervention results in an increased risk of suicide attempt compared to usual care. While unlikely, a clear finding of increased risk would certainly justify early termination.

### Appropriate level of external review

Monitoring trial integrity and participant safety is an obligation of investigators, other study personnel, and external safety monitors. While NIH guidance emphasizes the need for independent review of trial conduct [4], review by an external DSMB may not always be necessary. Many questions may require review by an independent monitor or safety officer, but only a smaller list of questions require confidential review of unblinded data by an independent board.

Independent review is needed for questions regarding potential harms of study interventions and adequacy of actions to protect participants from harm or address urgent clinical needs. Independent review may also be necessary to assess adequate rate of study enrollment, adequacy of intervention quality or fidelity, and quality or integrity of study data. In most cases, however, addressing these questions does not require any comparison between treatment groups. Consequently, there is no need for confidential review of unblinded data by a DSMB. Review by an independent safety officer with appropriate clinical expertise is often sufficient.

An external DSMB is, however, generally necessary for any confidential review of interim analyses to detect benefit, harm, or futility. Results of such analyses should be concealed from investigators and study team members [6, 20]. As discussed above, formal interim analyses requiring DSMB review are less often necessary for pragmatic trials than for explanatory clinical trials. A reviewing DSMB should have adequate statistical expertise to interpret interim analyses and adequate relevant clinical and policy expertise to weigh the consequences of premature termination to current participants, potential future participants, and other people living with the condition of interest. In pragmatic trials, expertise regarding health system data and operations will also be essential. DSMB members must understand real-world practice conditions and sources of error in real-world data.

### Summary and recommendations

The core ethical and regulatory obligations of pragmatic trial investigators do not differ from those of investigators leading more explanatory clinical trials. But specific features of pragmatic trials may require different procedures to satisfy those obligations. Table 2 lists features of pragmatic trials that may influence data and safety monitoring plans.

**Table 2:** Design features of pragmatic trials that influence data and safety monitoring plans

Design features intended to increase generalizability and/or efficiency	Effect on data and safety monitoring procedures
Enrollment of all eligible patients in a defined clinical population	Recruitment or enrollment rate is more predictable More vigorous advertising or outreach is less likely to increase enrollment
Cluster-level randomization	Statistical power is influenced by within-cluster correlation of outcomes Monitoring enrollment may include monitoring of cluster size and within-cluster correlation
Safety profiles of study treatments may already be established	Between-group comparisons to establish safety or risks may be less useful
Treatments often delivered by community providers, with little direct involvement by investigators	Fewer data available regarding adverse events or relationship between events and study treatment Investigators may have less ability to monitor or assure safe delivery of study treatments
Frequency of contact with study staff may vary between treatment groups	Comparison of adverse event rates between treatment groups may be biased
Fidelity of or adherence to treatments may be variable	Relationship between adverse events and study treatments may be more difficult to evaluate Valid inference may require some monitoring of (and corrective actions to improve) fidelity or adherence
Potential adverse events often ascertained from health system records	Identification of adverse events may be significantly delayed
Study outcomes often ascertained from health system records	Changes in health system record-keeping may affect integrity of study data Delays in access to records data may interfere with interim analyses of study outcomes
Study questions may involve wider range of outcomes, such as effects on health service use or cost	Longer follow-up periods may be necessary to assess some “downstream” outcomes of study treatments
Study questions may focus on implementation or policy, rather than individual clinical decisions	Decision thresholds for early termination of enrollment or intervention delivery may be asymmetric with respect to evidence for benefit or harm

While pragmatic trial departures from traditional explanatory trial design are intended to increase relevance and generalizability of findings, they also influence how investigators and independent monitors can monitor participant safety and assure trial integrity. Consequently, we recommend that investigators developing data and safety monitoring plans for pragmatic trials not simply replicate the monitoring procedures, analyses, and reports typically used in explanatory clinical trials. Instead, investigators should return to the core questions outlined in the original NIH policy:

- Will this trial enroll a sufficient sample of participants appropriate to answer the primary question?
- Do adverse events among trial participants indicate that study interventions involve unacceptable risk or harm?
- Will the quality or fidelity of treatment delivery be adequate for a valid test of the primary question?
- When an urgent clinical need is identified, do investigators and study staff consistently place duty to protect participant welfare over duty to the study protocol?
- Are outcome data of sufficient quality and consistency to yield a credible result?
- Do interim results clearly demonstrate that continuation of enrollment would be futile?
- Do interim results clearly demonstrate that continuing enrollment or intervention delivery would expose participants to unnecessary harm or deprive participants of a treatment now proven superior?

Plans for monitoring, interim analyses, reporting, and (if appropriate) premature termination should clearly address each of these specific questions.

## Data Availability

Not applicable.
